# Electrochemical Biosensing of Dopamine Neurotransmitter: A Review

**DOI:** 10.3390/bios11060179

**Published:** 2021-06-03

**Authors:** Sophie Lakard, Ileana-Alexandra Pavel, Boris Lakard

**Affiliations:** Institut UTINAM, UMR CNRS 6213, University of Bourgogne Franche-Comté, 16 Route de Gray, 25030 Besançon, France; sophie.lakard@univ-fcomte.fr (S.L.); ileana-alexandra.pavel-licsandru@univ-fcomte.fr (I.-A.P.)

**Keywords:** biosensors, dopamine, neurotransmitters, biomaterials, electrochemistry, microelectrodes

## Abstract

Neurotransmitters are biochemical molecules that transmit a signal from a neuron across the synapse to a target cell, thus being essential to the function of the central and peripheral nervous system. Dopamine is one of the most important catecholamine neurotransmitters since it is involved in many functions of the human central nervous system, including motor control, reward, or reinforcement. It is of utmost importance to quantify the amount of dopamine since abnormal levels can cause a variety of medical and behavioral problems. For instance, Parkinson’s disease is partially caused by the death of dopamine-secreting neurons. To date, various methods have been developed to measure dopamine levels, and electrochemical biosensing seems to be the most viable due to its robustness, selectivity, sensitivity, and the possibility to achieve real-time measurements. Even if the electrochemical detection is not facile due to the presence of electroactive interfering species with similar redox potentials in real biological samples, numerous strategies have been employed to resolve this issue. The objective of this paper is to review the materials (metals and metal oxides, carbon materials, polymers) that are frequently used for the electrochemical biosensing of dopamine and point out their respective advantages and drawbacks. Different types of dopamine biosensors, including (micro)electrodes, biosensing platforms, or field-effect transistors, are also described.

## 1. Introduction

Dopamine is a neurotransmitter playing essential roles in the cardiovascular and central nervous systems. As such, high dopamine levels indicate cardiotoxicity leading to rapid heart rates, hypertension, and heart failure [1]. On the contrary, low levels of dopamine in the central nervous system are implicated as a major cause of several neurological diseases such as Parkinson’s disease, schizophrenia, Alzheimer’s disease, stress and depression [2]. Therefore, it is obvious that dopamine measurements are required to understand its biological functions and related biological processes and mechanisms. Analytical techniques such as enzyme assays, liquid chromatography, mass spectroscopy, or capillary electrophoresis are the main techniques used for measuring dopamine. If a technique such as high-performance liquid chromatography (HPLC) with tandem mass spectrometric (MS/MS) detection is a powerful technique for the quantitative determination of dopamine [3,4], its cost is high. That is why there is a real interest in developing specific and low-cost biosensors taking advantage of dopamine’s easiness to be oxidized at the surface of an electrode.

Moreover, electrochemical biosensors generate a fast and sensitive response, allow monitoring in real-time, and can be miniaturized enough to be implanted in living cells. However, several issues need to be resolved. First, biosensors must be able to give a sensitive response in the appropriate range of concentration (0.01–1 µM for a healthy individual and in the nanomolar range for patients with Parkinson’s disease). Second, the biosensors must be selective enough to discriminate dopamine from its interferents, such as ascorbic acid or uric acid, which undergo oxidation within the same potential window as dopamine.

The selectivity of the dopamine biosensor is especially important and challenging when studying real samples (human serum or blood) due to the complexity of the biological samples. Indeed, such matrix does not contain only commonly occurring interferents such as ascorbic acid or uric acid, but also other neurotransmitters and molecules. To perform electrochemical detection of dopamine in real samples, it is generally necessary to add a few steps to sample preparation to reduce the matrix effect [5,6]. For blood samples, centrifugation is necessary before dilution and analysis, while urine samples are only diluted before analysis. The extraction of analyte or interferents using selective modification materials can also be used to lessen the matrix effect. In addition, when biological samples such as cerebrally extracellular fluid are studied, extremely low volumes of the samples are available, and thus, it is necessary to handle them with utmost care. For such samples, the real-time in vivo analysis with miniaturized microelectrodes is preferable.

To overcome these difficulties, researchers are developing new sensing materials that can be deposited onto the surface of an electrode. These can, in turn, increase the electrocatalytic properties of the electrode to obtain a more sensitive and selective response to dopamine. The most popular strategies used for the modification of electrodes are: the deposition of nanomaterials acting as redox mediators; the deposition of compounds containing specific groups that can facilitate the charge transfer; the deposition of nanostructures with a high specific surface area to increase the sensitivity of the electrode.

**Figure 1:**
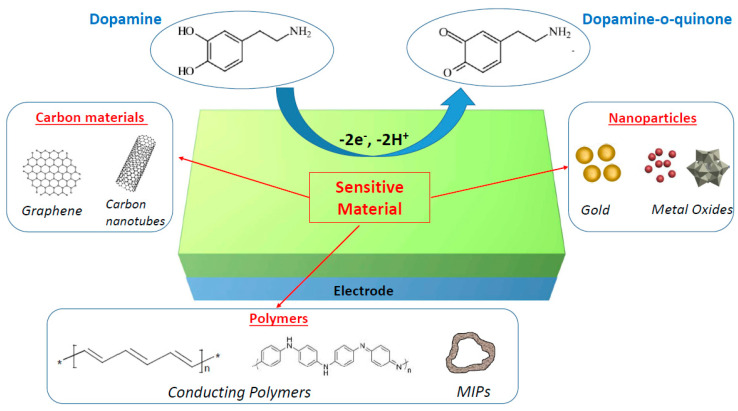
Schematic illustration of nanomaterial-modified electrodes for electrochemical biosensing of dopamine.

The main goal of the current review is to summarize the recent studies of nanomaterial-modified electrodes that were proven to be effective for the electrochemical detection of dopamine with high sensitivity and in a selective manner. Of the various nanomaterials available, we focused on three classes of materials, i.e., metal and metal oxide nanomaterials, carbon nanomaterials (graphene and carbon nanotubes), and polymers (conducting polymers and molecularly imprinted polymers), which can all improve the electrocatalytic oxidation of dopamine, as briefly described in Figure 1. This review should not be considered exhaustive due to the very high number of papers published on this topic, but we tried to select the most representative articles to illustrate the main strategies used to develop operational electrochemical dopamine biosensors.

## 2. Electroanalytical Methods

**Figure 2:**
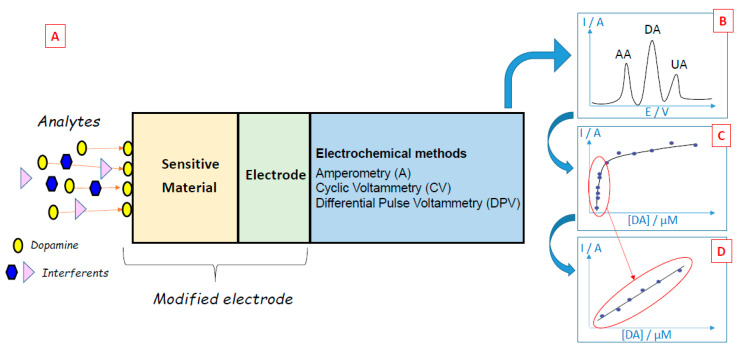
(**A**) Principles of dopamine electrochemical biosensors. (**B**) The typical voltammetric curve was obtained during the sensing of dopamine (DA) in the presence of ascorbic acid (AA) and uric acid (UA) interferents. (**C**) Typical evolution of the oxidation peak of dopamine as a function of dopamine concentration. (**D**) Typical calibration curve of dopamine.

When target biomolecules are captured by a sensing material deposited on the working electrode of an electrochemical biosensor, an analytical measurable signal is generated (Figure 2A). In the case of dopamine, many electrochemical methods (e.g., amperometry, cyclic voltammetry, differential pulse voltammetry) have been developed since dopamine can be oxidized easily [7], leading to the formation of dopamine-o-quinone through a two-electron process [8]. The electrons released by dopamine during its oxidation generate currents that may be linearly dependent on the concentration of the electroactive dopamine biomolecules, thus enabling the quantification of these compounds. Electrochemical methods have many advantages for dopamine detection: the low cost of electrochemical instrumentation, the size of the electrodes that can be conveniently implanted in living cells, the short response time, and the capacity to monitor dopamine in real-time. However, the detection of dopamine via electrochemical methods can be difficult when dopamine co-exists with other redox-active biomolecules that can be oxidized at close oxidation potentials, such as ascorbic acid or uric acid. To overcome this problem and perform selective detection, many materials have been developed and used to obtain selective modified electrodes.

### 2.1. Amperometry

In amperometric biosensors, the current produced during the oxidation or reduction of an electroactive biological element at a constant potential applied between a working electrode and a reference electrode is measured, providing specific quantitative analytical information [9,10,11]. These biosensors are inspired by the first amperometric biosensor developed by Clark in 1956, who fabricated an amperometric oxygen sensor producing a current proportional to the oxygen concentration when the potential was applied to a platinum electrode [12]. In the case of dopamine biosensors, a constant potential is applied, potential that is sufficient to oxidize dopamine to dopamine-o-quinone through a two-electron process. The current is proportional to the dopamine concentration over a more or less wide concentration range, thus allowing the quantification of the dopamine concentration in the sample [13,14,15]. However, amperometry is not selective since all electroactive compounds that can oxidize at the applied potential produce an amperometric response.

### 2.2. Cyclic Voltammetry (CV)

During cyclic voltammetry experiments, a current is produced by sweeping the potential applied between two electrodes over a range that is associated with the redox reaction of the analyte. This redox reaction generates a change in peak current that can be correlated to the concentration of the analyte, thus leading to specific quantitative analytical information [9,16,17]. This method has the advantage of providing both qualitative information deduced from the potential location of the current peak and quantitative information deduced from the intensity of the peak current.

For example, the oxidation of dopamine using cyclic voltammetry leads to an oxidation peak, which is characteristic of this biomolecule. By studying the evolution of the intensity of the oxidation peak present in the cyclic voltammograms for different dopamine concentrations, it is possible to draw calibration curves and quantify this compound (Figure 2C,D).

Fast-scan cyclic voltammetry (FSCV) is a derivative technique that can be used to address analytical challenges arising from biomedical needs to measure locally low concentrations of neurotransmitters [18,19]. With the FSCV technique, it is possible to perform real-time detection of dopamine on the subsecond time scale thanks to scan rates (100 V/s or faster) that are 1000-fold faster than traditional cyclic voltammetry [20]. FSCV can only be achieved at a microelectrode having a small-time constant for fast capacitive charging [21]. In addition, carbon-fiber microelectrodes are generally used since they have surface oxide functional groups that adsorb cations [22]. Recent progress has been done concerning waveform optimization, electrode development, and data analysis [23], which accelerated the research in the field of dopamine detection and monitoring.

The monitoring of dopamine by FSCV has been exhaustively described by Venton and Cao [24]. It consists of applying a holding cathodic potential of −0.4 V to the working carbon microelectrode to selectively preconcentrate cationic dopamine on the microelectrode surface. Then, a triangular waveform, with a fast-scan rate, is applied repeatedly to scan the electrode to a switching anodic potential of +1.3 V and back to oxidize dopamine and reduce dopamine-o-quinone. After that, the background current is subtracted out from the overall CV to get the background-subtracted CV, which has a unique shape for each electroactive compound.

### 2.3. Differential Pulse Voltammetry (DPV)

Differential pulse voltammetry is a derivative of the linear sweep voltammetry technique with a series of regular voltage pulses superimposed on the potential linear sweep [25,26,27]. In DPV, a base potential value is chosen at which there is no faradaic reaction, and this potential is applied to the electrode. The base potential is increased between pulses with equal increments. The current is measured just before each potential change, and the current difference is plotted against the potential. Sampling the current immediately before the potential is modified reduces the effect of the charging current. In the linear sweep technique, an oxidation process leads to the formation of a wave in the voltammogram, but in the DPV technique, an oxidation process originates a peak. This sharper shape facilitates the interpretation of the voltammogram and renders DPV more accurate than linear voltammetry. This is particularly useful in the case of electrochemical biosensors since it is easier to discriminate the sharp peaks attributed to dopamine, ascorbic acid or uric acid when applying the DPV technique compared to the large waves obtained by linear voltammetry (Figure 2B).

## 3. Electrodes and Microelectrodes

Neurotransmitter biosensors and neural-electrical interface devices are being more and more developed for monitoring the release of neurotransmitters in vivo and restoring or supplementing the function of the nervous system, respectively. For this purpose, microelectrodes are of great interest to monitor locally the fluctuations of the neurotransmitter concentrations [22,28] and record or stimulate large populations of neurons [29,30,31]. Recent developments in nano and microtechnology enable the manufacture of such micro or ultramicroelectrodes (called UMEs) and microfabricated electrode arrays (called MEAs), whose use is not restricted by any particular electrochemical techniques and can be used both in vivo and in vitro. Such miniaturized electrodes allow to perform very local measurements, improve the signal-to-noise ratios, obtain faster response times, and decrease the impact of the iR drop [28]. A wide variety of materials have been used for the manufacture of microelectrodes, but the most used electrode materials are noble metals usually, platinum [32,33] or gold [34,35,36,37,38,39], and carbon materials [40,41,42,43,44,45,46,47,48], even some metal oxides [49,50] or semiconductors [51,52] can also be used.

Metal electrodes are used due to their high electrical conductivity and to the ease of fabrication of metal microelectrode arrays using microsystems technology [36]. In particular, microfabricated electrode arrays composed of tens or hundreds of metallic electrodes can be easily fabricated to perform multi-analyte monitoring [36,37,38]. Gold surface is also of interest since it can be easily functionalized by the formation of covalent bonds with thiol groups, thus enabling the formation of self-assembled monolayers that can act as a sensitive layer of the dopamine biosensor [39].

However, carbon-fiber electrodes are the most widely used for the electrochemical characterization of dopamine oxidation [40,41] because they possess numerous advantages. Indeed, carbon-fiber electrodes are biocompatible and not toxic to cells [42], they can be easily miniaturized (they are usually less than 10 µm in diameter), allowing very local measurements [43], they possess good electrochemical properties leading to a very high sensitivity compared to other electrodes [44], and they are relatively not susceptible to fouling by-products of dopamine electro-oxidation [22].

In addition, carbon-fiber electrodes can be functionalized or modified in order to increase their selectivity for cationic dopamine over anionic interferents such as ascorbic acid. Thus, a Nafion coating can be deposited on carbon electrodes to repel negative interferents since Nafion is an anionic polymer [45]. Similarly, carbon electrodes can be modified with negative 4-sulfobenzene [46]. It is also possible to incorporate carbon nanotubes in such carbon-fibers electrodes in order to increase the sensitivity due to the electrical conductivity and high specific surface area of the nanotubes [47]. By combining these two approaches, carboxyl groups were used to modify carbon nanotubes, and the resulting nanotubes were incorporated into carbon electrodes leading to a high sensitivity and electron transfer kinetics [48].

Carbon-fiber microelectrodes are also used to perform single-cell amperometry measurements, which allows monitoring the release of electroactive molecules, such as dopamine from individual vesicles, since they can be positioned in the close proximity of a cell thanks to their small size [53,54]. As a constant and appropriate potential value is applied for oxidizing the molecules released by exocytosis, the exocytotic activity of the cell can be monitored in real-time and displayed as a succession of amperometric spikes. In this context, each amperometric spike corresponds to an individual exocytotic event. Therefore, the frequency of spikes reveals the activity of the cell in the close vicinity of the electrode surface since the integration of the spikes gives the total charge transferred during the event, which allows by Faraday’s law the quantification of the number of moles released. In addition, the detailed analysis of each spike gives access to the dynamics of the secretory events and the number of neurotransmitters discharged. In addition, Ewing’s group succeeded in counting catecholamine molecules in individual nanometer transmitter vesicles by combining resistive pulse measurements in a nanopore pipet and vesicle impact electrochemical cytometry (VICE) at an electrode as the vesicles exits the nanopore [55]. The same group used open carbon nanopipettes with radius between 50 and 600 nm to control the translocation of different-sized vesicles through the pipette orifice followed by nanoelectrochemical analysis. VIEC was used to count catecholamine molecules expelled from single vesicles onto an inner-wall carbon surface. This, in turn, allowed the counting for vesicles in a living cell [56].

## 4. General Overview of Modification Materials for Dopamine Electrochemical Sensing

### 4.1. Metal and Metal Oxide Nanomaterials

#### 4.1.1. Metal Nanostructures

Metal nanoparticles (NPs) are an advantageous material for biosensing [57] owing to their excellent conductivity, signal amplification, and facilitation of both electron transfer and electrical contact between the redox center of a biomolecule and the electrode surface. These electrocatalytic properties of the metal nanoparticles are far superior to the bulk metals due to their high surface area and improved reactivity. In particular, gold nanoparticles have been used in various electrochemical biosensors since gold enhances cell adhesion and growth [58].

Thus, a nanostructured gold surface consisting of closely packed outwardly growing spikes showed a significant electrocatalytic effect for the electrooxidation of dopamine due to the presence of numerous surface-active sites [59]. The resulting biosensor exhibited a linear range of 1–100 μM, with a detection limit of 5 µM using differential pulse voltammetry (Table 1). In another study, nanostructured gold surfaces were prepared by electrodeposition and used for the determination of dopamine [60]. The square wave voltammetry peak current was linearly dependent on dopamine concentration up to 10 μM, with a detection limit of 0.57 μM.

Recently, another dopamine biosensor based on 3D nanocone-shaped gold nanostructures was fabricated to detect dopamine from neuronally differentiated rat phaeochromocytoma (PC12) cells. Cyclic voltammetry confirmed that the gold nanoelectrodes showed higher oxidation peaks towards dopamine than the bare nanoelectrode [61]. Furthermore, 3D cylindrical gold nanopillar pattern arrays were fabricated to detect dopamine leading to excellent sensing capabilities, with a detection limit of 5.83 μM, in the presence of interferents [62]. Additionally, an electrochemical platform containing numerous microsized gold pyramids was fabricated to increase the surface area and efficiently detect dopamine secreted from neuroblastoma cells [63]. The limit of detection was 0.5 nM, and the wide linear range was from 10 nM to 500 μM. Another electrode modified by electrodeposition of gold nanoparticles in order to increase the electron transfer during dopamine detection was reported [64]. This modified electrode allowed the detection of dopamine released from living PC12 cells through amperometry.

Palladium nanoparticles were also used to modify electrodes, which displayed good electrochemical catalytic activities towards dopamine since the calibration curve obtained using differential pulse voltammetry was in the range of 0.5–160 μM, and the detection limit was 0.2 µM [65]. Some dopamine electrochemical biosensors incorporated bimetallic nanoparticles were used to enhance electron transfer due to the wide surface areas. Thus, Au–Pt nanoflowers were used, and the amperometric results showed a large linear range of dopamine detection from 0.5 μM to 0.18 mM with a limit of detection of 0.11 μM [66]. Similarly, Au–Pt bimetallic nanoparticles were deposited on sulfonated nitrogen sulfur. The limit of detection and linear range for dopamine, as determined by differential pulse voltammetry, were 0.006 μM and 1–1000 µM [67].

Although metal electrodes have many advantages, they could suffer from a lack of selectivity. To address this issue and enhance selectivity, a strategy consists of incorporating aptamers, which are well-known small oligonucleotides capable of binding to a specific target of interest, can be employed. Thus, by combining the high conductivity and specific surface area of metal nanoparticles with the selectivity of aptamers, it is possible to obtain very efficient biosensors. An electrode modified with gold nanostars and a dopamine-binding aptamer exhibited a very low detection limit of 0.0019 nM. The presence of many interferents did not hinder the sensitivity of the biosensor due to the specific binding between the aptamers and the dopamine molecules [68]. Similarly, a platform composed of a spindle-shaped gold nanostructure and dopamine-binding aptamers showed high selectivity, high stability, and a limit detection of 0.01 nM using differential pulse voltammetry [69]. As can be deduced from these two latter studies, it can be relevant to combine metal nanoparticles, having high electrical conductivity and high specific surface area, with other materials that can offer additional benefits such as selectivity. In the subsequent sections, many dopamine biosensors using metal nanoparticles in combination with other materials (carbon, polymers, etc.) will be described.

**Table 1:** A comparison of the performances of electrochemical dopamine biosensors based on metal nanomaterials.

Active Layer	Linear Range	Detection Limit	Reference
Au nanostructures (spikes)	1–100 µM	5 µM	[59]
Au nanostructures	1–10 µM	0.57 µM	[60]
Au nanostructures (cones)	1–43 µM	0.184 µM	[61]
Au nanopillars	1–100 µM	5.83 µM	[62]
Au nanopyramids	10 nm–500 µM	0.5 nM	[63]
Pd NPs	0.5–160 µM	0.2 µM	[65]
Au–Pt nanoflowers	0.5 µM–0.18 mM	0.11 µM	[66]
Au–Pt NPs	1 µM–1 mM	6 nM	[67]
Au nanostars—dopamine aptamer	1–100 ng/L	0.29 ng/L	[68]
Au nanostructures—dopamine aptamer	25 ng/L–3 µg/L	2 ng/L	[69]

#### 4.1.2. Metal Oxide Nanostructures

Metal oxide nanoparticles and nanostructures can also be used for electrochemical sensing of dopamine owing to their high surface area and good biocompatibility [70]. Thus, iron oxide and platinum were synthesized to prepare dumbbell-like FePt–Fe_3_O_4_ nanoparticles, which electrocatalyzed the oxidation and increased the sensing of dopamine, leading to a linear range of 0.1–90 µM and a detection limit of 1 nM [71] (Table 2). They were also used successfully to monitor the dopamine released from PC12 cells stimulated with K^+^ (extracellular K^+^ is very frequently used to induce a release of positively charged dopamine molecules by living cells). Another electrochemical sensor based on NiO-lacy flower-like geometrical structure with semi-spherical head surfaces associated with abundant and well-dispersed tubular branches with needle-like open ends was developed [72]. This geometry possessed many catalytic active sites and favors the sensing of dopamine, leading to a detection limit of 85 nM and a linear range of 0.5 μM–5 μM. Ultrasensitive in vitro monitoring of dopamine released from PC12 cells was also realized, showing that these structures could be used for clinical diagnosis.

A flake-like MoS_2_-modified electrode was used as an electrochemical sensor and exhibited higher electrocatalytic activity in the oxidation of dopamine (in terms of higher oxidation peak current and lower oxidation potential) when compared with bare electrode [73]. The flakes-like MoS_2_ led to a wide linear response range (0.006–181 μM) and a very low detection limit of 2 nM. Another photoelectrochemical sensor, including a nanoMoS_2_-modified gold electrode, displayed a very high sensitivity with a limit of detection of 2.3 pM in the presence of other interfering molecules, such as ascorbic acid, uric acid, and cysteine [74]. Hierarchically nanostructured ZnO flowers composed of bundled nanochains were also synthesized and showed both good sensitivity and selectivity for the detection of dopamine (detection range: 0.1–800 µM, detection limit: 60 nM) [75].

Highly ordered mesoporous Fe_3_O_4_ materials were synthesized by using mesoporous silica as a hard template [76]. The ordered mesoporous Fe_3_O_4_ with high surface area modified glassy carbon electrode showed a high catalytic activity to dopamine with a detection limit of 0.8 nM and a linear range between 2 and 600 nm. The dopamine released from cultured PC12 cells was also measured in real-time using amperometry. Similarly, highly ordered mesoporous ZnFe_2_O_4_ was prepared via a nanocasting method and was found to be highly sensitive in the electrochemical detection of dopamine in a wide linear range from 2 to 600 nM and a low detection limit of 0.4 nM [78]. It was also successfully used to monitor the increase in dopamine concentration induced by K^+^ stimulation of living PC12 cells in a neurological environment.

Some examples were given here describing dopamine biosensors based on metal oxide nanostructures. However, if metal oxides have the advantages of high surface area and biocompatibility, they have the disadvantage of low conductivity. That is why they are often combined with more conducting materials, in particular carbon materials, to obtain more efficient biosensing of dopamine, as it will be shown in subsequent sections.

### 4.2. Carbon Materials

Carbon materials are used in various fields due to their thermal stability, chemical resistance, and excellent mechanical properties [78,79,80]. They have tremendous potential for sensing target biomolecules, such as dopamine, due to their excellent electrical conductivity, fast electron transfer kinetics, and reasonable biocompatibility [81,82]. Carbon materials are also low-cost nanomaterials that can be used alone or in combination with other materials.

#### 4.2.1. Carbon Nanotubes

Carbon nanotubes (CNTs) are grouped into two sorts: cylinder-shaped single-walled CNTs (SWCNTs) with diameters in the range of a nanometer, and multi-walled CNTs (MWCNTs), consisting of nested single-wall CNTs weakly bound together by van der Waals interactions. Individual CNT walls can be metallic or semiconducting depending on the alignment of the graphene lattice with respect to the tube axis. CNTs have been widely used as electrode modifiers in electrochemical biosensing [83] and showed good electrocatalytic properties in the oxidation and the reduction of many different compounds owing to their high electrical conductivity [84], the possibility to be functionalized, the large specific surface area that allows immobilization of receptor moieties for biosensing applications, and good mechanical and chemical stability [85].

NCTs are frequently used to decrease the oxidation potential of dopamine and facilitate its biosensing. For example, SWCNTs and MWCNTs were introduced in a carbon electrode modified with 5-amino-3′,4′-dimethyl -biphenyl-2-ol. The oxidation of dopamine occurred at a potential about 170 mV (resp. 160 mV) less positive than that of the electrode, which did not contain CNTs, thus leading to a linear response over a wide concentration of dopamine (1.2–900 µM with SWCNTs and 1.2–800 µm with MWCNTs, with a detection limit of 0.57 µM with SWCNTs and 0.16 µM with MWCNTs) [86,87] (Table 3).

It is also possible to incorporate negatively charged SWCNTs into carbon electrodes. For example, negatively charged SWCNTs, obtained by the introduction of carbonyl functionalities, selectively attracted the cationic dopamine towards the electrode and repelled the anionic ascorbate and uric acid coexisting in the same solution (detection limit: 15 nM) [88]. Similarly, SWCNTs modified with sodium dodecyl sulfate possessed a negative charge that allowed a successful determination of dopamine in the presence of ascorbic acid and uric acid and showed good recovery in some biological fluids. The catalytic peak currents obtained by voltammetry increased linearly with the increase in dopamine concentrations in the range of 5–100 µM with a detection limit of 200 nM [89].

SWCNTs can also be combined with Nafion and poly(3-methylthiophene) to obtain synergetic effects since Nafion has high antifouling capacity and permeability to cations when SWCNTs and conducting polymers have high electrocatalytic properties. The resulting electrode led to the oxidation of dopamine, uric acid and ascorbic acid at distinguishable potentials to form well-defined and sharp peaks, making possible simultaneous determination of each species. Moreover, the proposed electrode was advantageously employed for the determination of dopamine in real pharmaceutical and clinical formulations [90]. Another electrochemical sensor included SWCNTs dispersed in chitosan and treated by ionic liquids allowed the determination of dopamine for concentration between 0.5 and 30 μM within real samples (detection limit: 0.16 μM). In this case, the use of SWCNTs and an ionic liquid increased significantly the anodic peak current intensity, thus facilitating the detection of the neurotransmitter [91]. Additionally, a sensing platform for the detection of dopamine used CNTs modified with overoxidized polypyrrole that increased the sensitivity and electroactivity of the sensors, leading to a limit of detection of 136 pM. This platform was also used to cultivate dopaminergic neurons and a specific amperometric current of dopamine released from the cells was detected through electrochemical experiments [92].

MWCNTs can also be modified with nanoparticles. Thus, a biosensor allowing the monitoring of extracellular dopamine in neuronal cells was developed, combining MWCNTs and Ag–Au nanoparticles in the same electrode. The presence of metal nanoparticles increased the electron transfer rate and the sensing performances of the sensor, leading to a linear range of 3 nM–2.3 µM and a detection limit of 0.23 nM [93]. Similarly, another biosensor permitted the monitoring of extracellular dopamine in neuronal cells by combining MWCNTs with graphene nanoparticles that increased the surface area of the sensor leading to a linear range of 5 nM–100 µM and a detection limit of 0.87 nm [94]. Additionally, MWCNTs–MoS_2_-decorated cobalt oxide polyhedrons were synthesized. The resulting composites possessed good porosity, large electrochemical area, roughened surface, and excellent electrocatalytic ability as proven by the limit of detection (13 nM) of the dopamine biosensor, which was successfully tested in rat brain and human blood serum samples [95].

#### 4.2.2. Graphene

Graphene is a 2D nanomaterial consisting of a single layer of sp^2^ network of carbon atoms. Graphene and its derivatives graphene oxide (GO) and reduced graphene oxide (rGO) have emerged as promising materials for wide practical applications, including electrocatalysis and electroanalysis, due to their beneficial characteristics, including high thermal and electrical conductivity [96], excellent mechanic performances [97], a wide electrochemical potential window (2.5 V), and the possibility to be easily functionalized by covalent or non-covalent binding or modified with elemental dopants [98]. In addition, graphene possesses several specific advantages for dopamine electrochemical detection since: (i) dopamine molecules are capable of thermodynamic adsorption on a graphene surface through π–π stacking interactions, (ii) graphene possesses a very high specific surface area that can support the adsorption and diffusion processes of dopamine, and (iii) the presence of oxygen-containing groups on the GO or rGO surface accelerates the electron transfer during electrochemical biosensing and generally leads to a better selectivity, sensitivity, and limit of detection [99].

The addition of graphene or (reduced) graphene oxide is sufficient to observe an increase in the response of dopamine sensors and to obtain a wide linear range and low detection limit: 10 nM–100 µM and 1 nM for graphene using cyclic voltammetry [100], 4–100 µM and 2.6 µM for graphene using differential pulse voltammetry [101], and 1–80 µM and 0.46 µM for reduced graphene using cyclic voltammetry [102] (Table 4).

Poly(3,4-ethylenedioxythiophene) (PEDOT) is an excellent conducting polymer for electrochemical detection of dopamine due to its low oxidation potential, and it can be easily deposited onto graphene. Such modified electrodes provided a low detection limit of 0.33 µM and an excellent peak separation between dopamine and its interferents [103]. Polypyrrole (PPy) is another conducting polymer that can be combined with graphene [104] or graphene oxide [105] to obtain efficient dopamine sensors with a linear range of 0.5 µM–10 µM and 1 µM–150 µM, respectively, and a limit of detection of 0.1 µM and 0.02 µM, respectively. Other electrodes, modified by polyvinylpyrrolidone (PVP) and graphene, exhibited a very low detection limit of 0.2 nM thanks to the strong adsorption of PVP to phenolic compounds due to hydrogen bonding between the imide of the PVP and OH group in dopamine [106].

Metal oxides are also frequently combined with graphene to obtain modified electrodes. Thus, the carbon electrodes modified with core–shell Fe_3_O_4_-graphene nanospheres coated with Nafion exhibited excellent electrocatalytic activity towards the oxidation of dopamine [107]. Under the optimal conditions, the broad linear relationship was obtained from 0.020 μM to 130.0 μM with the detection limit of 7 nM. Similarly, the Cu_2_O–rGO nanocomposite was used for dopamine determination by cyclic voltammetry [108]. The results showed that the oxidation peaks of ascorbic acid, dopamine, and uric acid were well-separated, and the detection limit of 6 nM was achieved. Other oxides, such as Mn_3_O_4_ [109] or NiO [110], have also been combined with graphene, but their sensing abilities were less interesting.

A graphene–nickel hydroxide modified carbon electrode exhibited an appreciable electrocatalytic effect for the simultaneous detection of lower concentrations of dopamine compared to graphene modified carbon electrode [111]. The detection limit attained by differential pulse voltammetry was 120 nM. In another work, heterostacked layers, including rGO and layered double hydroxides, were used as sensitive layers [112]. The electrochemical peaks of ascorbic acid, uric acid, and dopamine were well-discerned by voltammetry, and a low detection limit of 0.1 nM was obtained. Moreover, the rGO-based composite was utilized to cultivate neuroblastoma cells and to quantify the amount of dopamine released from the living cells, thus demonstrating its biocompatibility.

Metal nanoparticles can be combined with graphene and enhance its electrocatalytic properties. Thus, an electrochemical sensor was prepared by modifying an electrode with rGO and gold nanoparticles having great conductivity and a large surface area [113]. The results showed that it exhibited linearity in the range of 0.1–100 μM of dopamine and the limit of detection was 0.098 μM. Better sensing properties were obtained using an electrochemical sensor based on ionic liquid functionalized graphene oxide supported gold nanoparticles coated onto a carbon electrode. This was possible since ionic liquids can be uniformly dispersed in graphene oxide owing to their good solvation properties [114]. Indeed, this hybrid nanomaterial showed excellent electrocatalytic activity towards dopamine. Under the optimum conditions, differential pulse voltammetry was employed to detect ultra-trace amounts of dopamine (limit of detection 2.3 nM) for a wide linear range of 7–5 μM. In another study, an organic field-effect-transistor biosensor using platinum nanoparticle-decorated rGO immobilized on a graphene substrate by π–π interactions was developed [115]. It showed high sensitivity to remarkably low dopamine concentrations since its limit of detection was 10^−16^ M and selectivity among interfering molecules. In another study, the rGo composite was modified with dendritic platinum nanoparticles, which increased the sensitivity of sensing dopamine released from pheochromocytoma cells (detection limit: 5 nm, linear range: 87–100 μm) [116], the biosensing platform was used both for the cultivation of living cells and for the detection of dopamine secreted by these cells.

**Table 4:** A comparison of the performances of electrochemical dopamine biosensors based on graphene and its derivatives.

Active Layer	Linear Range	Detection Limit	Reference
Graphene	10 nM–100 µM	1 nM	[100]
Graphene	4–100 μM	2.6 µM	[101]
Reduced graphene	1–80 μM	0.46 µM	[102]
PEDOT–Graphene	1–150 µM	0.33 µM	[103]
PPy–Graphene	0.5–10 µM	0.1 µM	[104]
PPy–Graphene oxide	1–150 µM	0.02 µM	[105]
PVP–Graphene	0.5 pM–1.13 mM	0.2 nM	[106]
Fe_3_O_4_–Graphene	0.02–130 μM	7 nM	[107]
Ni(OH)_2_–Graphene	0.44–3.3 μM	120 nM	[111]
Cu_2_O–Graphene oxide	0.01–1 µM	6 nM	[108]
Zn–NiAl–Graphene oxide	1 nM–1 µM	0.1 nM	[112]
Au NPs–Graphene oxide	0.1–100 μM	0.098 μM	[113]
Ionic liquid–Au NPs-Graphene oxide	7 nM–5 μM	2.3 nM	[114]
Pt NPs–Graphene oxide	87 nM–100 μM	5 nM	[116]
Pt NPs–Graphene oxide (FET)	1 pM–0.1 µM	10^−4^ pM	[115]

### 4.3. Polymer Materials

#### 4.3.1. Conducting Polymers

Conducting polymers are very common modifiers that can be chemically or electrochemically deposited over bare electrodes from their monomer solutions. They have an extended π-conjugated structure with alternating single and double bonds across the polymeric chain, which causes delocalization of the electrons in the polymeric backbone, and is responsible for their outstanding electrical and optical properties. In particular, conducting polymers can reach excellent electrical conductivity comparable to the one displayed by metals. In addition, they present other advantages for use in electrochemical biosensing, such as the possibility to be easily modified with functional groups or with nanoparticles, such as metal oxide nanoparticles [117] and metal or carbon nanostructures [118], the formation of a protective layer to avoid surface fouling, high biocompatibility, the possibility to be selective towards target bioanalytes by avoiding the interfering species through hydrophobic, hydrophilic, ion-exchange, or electrostatic interactions [5,6]. For all these reasons, conducting polymers and their nanocomposites are widely used for biomedical applications [119], in particular for electrochemical sensing of biomolecules [9,120], including neurotransmitters such as dopamine [121].

Polypyrrole is one of the most widely used polymers for biosensing due to its good redox activity, water solubility, and biocompatibility, as well as ease of modification and ability to form nanostructures. Thus, polypyrrole nanofibers were deposited on modified electrodes by electropolymerization. The resulting sensor showed high selectivity to dopamine with a low detection limit of 7 nM thanks to the high reactivity due to the large surface area of the nanofibers [122] (Table 5). In another study, an electrode was modified by electropolymerization of a pyrrole derivative (pyrrole-3-carboxylic acid) [123]. The polymer film displayed superior electron transfer characteristics according to the bare electrode, and the ionized carboxyl groups showed high affinity towards positively charged dopamine. As a consequence, the modified electrode exhibited linear responses for dopamine concentration values going from 0.025 to 7.5 µM, and the detection limit was determined as 2.5 nM. It also showed high selectivity towards dopamine by discriminating its oxidation potential from the common interfering substances. Using the same strategy, many other dopamine biosensors have been fabricated using carbon electrodes modified by electrodeposited conducting polymers such as poly-4-amino-6-hydroxy-2-mercaptopyrimidine [124], poly-5-amino-1H-tetrazole [125], poly(cinnamic acid) [126], poly(eriochrome black T) [127], poly(safranine O) [128], or poly(trypan blue) [129].

Another biosensor using electropolymerized poly (2-napthol) orange film deposited on a carbon electrode is worth considering due to the importance of charges and interactions in the sensing mechanism [130]. Indeed, it was shown that the oxidation of dopamine was promoted by both the hydrogen bond formation between hydroxyl groups of dopamine and sulfonate groups of the polymer and by the electrostatic attractive interactions between positively charged amino groups of dopamine and negatively charged sulfonate groups of the polymer, leading to the low limit of detection of 95 nM. Similarly, the interactions between sulfonate anions and charged dopamine were used in other works [131,132]. An electrode was modified by poly(1,5-diaminonaphthalene) and reactive blue-4 dye-containing sulfonate anions to detect positively charged dopamine in human blood serum and urine samples (concentration range: 5.0–100 μM, detection limit: 0.1 μM) [131]. Using the same strategy, interdigitated gold microelectrodes were coated with electropolymerized polypyrrole doped with polystyrene sulfonate anions [132]. The polymer-modified electrodes were used to amperometrically detect dopamine released by populations of differentiated PC12 cells upon triggering cellular exocytosis with an elevated K^+^ concentration. A comparison between the generated current on bare gold electrodes and polypyrrole-modified electrodes illustrated the clear advantage of the modification, yielding 2.6-fold signal amplification. It is also possible to incorporate a mediator such as a ferrocene in a conducting polymer film, such as PEDOT, to enhance the electron transfer between the modified electrode and dopamine biomolecules, hence facilitating the biosensing of the neurotransmitter. The resulting biosensor presented a linear range between 0.01 and 0.9 mM, and a limit of detection of 1 µM [133].

Several works have been dedicated to the use of electrodes modified by a conducting polymer and gold nanoparticles to detect dopamine. For example, an electrode was modified by gold nanoparticles, poly(3,4-ethylenedioxythiophene) and sodium dodecyl sulfate [134]. This modified electrode showed a higher catalytic activity due to the rich electron cloud in the polymer and electrocatalytic properties of the nanoparticles and sodium dodecyl sulfate. The dopamine concentration could be measured in the linear range of 0.5–140 μM, with a low detection limit of 0.39 nM. The modified electrode was validated for the determination of dopamine in human urine. Another electrode was modified by electrodeposition of gold nanoparticles over polyaniline using linear voltammetry leading to an efficient loading of nanoparticles in the polymer matrix [135]. The resulting electrode showed enhanced electrocatalytic activity in the working linear range of 20–100 μM and a detection limit of 16 μM. Such enhanced electrocatalytic response was attributed to a synergistic interaction between the polymer film and the nanoparticles. Au–PANI core–shell nanocomposites were prepared by one-step chemical oxidative polymerization of aniline using chloroaurate acid as the oxidant and AuNPs as the seeds and then deposited onto glassy carbon electrodes for the determination of dopamine using differential pulse voltammetry [136]. The π-rich nature of polyaniline, the π–π interaction between the phenyl ring of dopamine and the polyaniline promoted the influx of dopamine molecules to the electrode surface. The catalytic peak currents were linear, with the concentration of dopamine in the range of 10–1700 μM, and the detection limit was 5 μM.

Composites were also prepared from conducting polymers and carbon nanomaterials. For example, a nanocomposite composed of poly (3,4-ethylenedioxythiophene) doped with graphene oxide was electrodeposited on an electrode and exhibited lowered electrochemical impedance and excellent electrocatalytic activity towards the oxidation of dopamine (wide linear range from 0.1 to 175 μM, with a detection limit of 39 nM, no interference with uric acid and ascorbic acid) due to the large surface area, which provided many active sites that accelerate the electron transfer process of dopamine [137]. An electrochemical aptasensor based on graphene–polyaniline nanocomposites film for dopamine determination was reported [138]. The resulting biosensor exhibited a good current response for dopamine determination, good electron transfer activity, and high conductivity due to the synergetic effect of graphene and polymer. The electrochemical aptasensor showed a linear response to dopamine in the range of 0.007–90 nM and a limit of detection of 0.002 nM. It was also successfully tested on human serum samples. To measure the amount of dopamine released, an array of neural microelectrodes coated with graphene–polypyrrole nanocomposites was fabricated [139]. The deposited graphene significantly increased the surface area of the modified electrode, leading to an excellent selectivity, sensitivity, linearity of the response in the range of 0.8–10 μM, and low detection limit (4 nM) to dopamine. Furthermore, the nanoelectrode combined with the patch-clamp system was used to detect quantized dopamine release from PC12 cells under 100 mM K^+^ stimulation.

Several biosensors based on conducting polymers and carbon nanotubes have been developed. For example, electrodeposited poly(3,4-ethylenedioxythiophene) doped with pure CNTs were deposited on a carbon paste electrode leading to a sensitive dopamine biosensor with excellent catalytic property, a linear range from 0.1 to 20 μM, and a detection limit of 20 nM [140]. Similarly, another sensitive electrochemical sensor was obtained by electropolymerization of cystine on a carbon electrode followed by drop-casting of CNTs, leading to good electroactivity towards the biosensing of dopamine (linear range: 10–200 µM, limit of detection: 2.8 µM) [141]. An original biosensor that did not rely on direct oxidation of dopamine was also developed [142]. The sensitive layer consisted of poly(anilineboronic acid)–carbon nanotube composite and utilized the excellent permselectivity of Nafion. Thanks to the high-affinity binding of dopamine to the boronic acid groups of the polymer deposited on the carbon nanotubes, a significant improvement in the electrochemical detection properties of dopamine was achieved. The high sensitivity and selectivity of the sensor (linear range: 1–10 nm, the limit of detection: 0.016 nm) showed excellent promise towards molecular diagnosis of Parkinson’s disease. A complex sensitive layer made of MWCNTs electrochemically deposited on glassy carbon, then modified with nanoceria–poly(3,4-ethylenedioxythiophene) composite was also prepared in the presence of sodium dodecylsulfate [143]. Compared with a bare electrode, the modified electrode exhibited a more effective electrocatalytic performance with regards to the oxidation of dopamine with a large linear range of 0.10–400 μM and a good detection limit of 0.03 μM. Moreover, no interference effects were observed in the presence of Ca^2+^, K^+^, Na^+^, glucose, urea, sucrose, citric acid, and cystine, and the sensor was successfully utilized in pharmaceutical samples.

The field-effect transistor (FET) is a type of transistor that uses an electrical field to control the flow of current. The electrical signals of a FET are generally expressed as the changes in current intensities. FET is currently one of the most popular types of devices in the biosensor field due to its extreme sensitivity [144,145]. Since the amount of dopamine exocytosed from living cells is extremely low, FET appears as a good choice owing to its high sensitivity and that is why several FETs were used recently to detect dopamine from living neurons. In addition, FET technology is compatible with conducting polymers due to their solution process ability and tunable properties. For example, an organic FET based on poly(3,4-ethylenedioxythiophene):poly(styrene sulfonic acid) (PEDOT:PSS) with different gate electrodes, including graphite, Au, and Pt electrode, have been used as dopamine sensor [146]. The sensitivity of this organic electrochemical transistor to dopamine was found to be dependent on its gate electrode and operation voltage, and the device with a Pt gate electrode characterized at the gate voltage of 0.6 V shows the highest sensitivity and lowest detection limit (less than 5 nM). Another PEDOT:PSS FET has been developed and used for the selective detection of dopamine in the presence of interfering compounds, and the selective response has been implemented using a potentiodynamic approach by varying the operating gate voltage and the scan rate [147]. The trans-conductance curves allowed to obtain a linear calibration plot for ascorbic acid, uric acid and dopamine and to separate the redox waves associated with each compound. Similarly, specific detection of dopamine was achieved with organic neuromorphic devices with no specific recognition function in an electrolyte solution [148]. The response to voltage pulses consisted of amplitude-depressed current spiking mimicking the short-term plasticity of synapses. Both the capacitance and the resistance of the PEDOT:PSS layer deposited on the device, changed with solution compositions, lead to the detection of dopamine from pM to mM range of concentrations.

Another FET was functionalized with carboxylated polypyrrole nanotubes/aptamer and used to detect the dopamine secreted by living cells [149]. A very low detection limit of 100 pM was obtained, as well as a linear range going from 0.1 nM to 10 μM and a high selectivity since catechol, epinephrine, and ascorbic acid did not interfere with dopamine sensing. Another biosensor using FET technology composed of immobilized conducting-polymer (3-carboxylate polypyrrole) particles decorated with Pt particles was used to detect dopamine [150]. These 60 nm polymer nanoparticles were stirred in PtCl_4_ aqueous solutions in order to induce a covalent bonding between the Pt^4+^ ions and the negative charge of the carboxylate group present in the polymer structure. Then, by the addition of NaBH_4_, Pt^4+^ ions were reduced to Pt nanoparticles. The resulting hybrid particles were then immobilized on an amine-functionalized interdigitated-array electrode substrate through the formation of covalent bonds with amine groups. The resulting Pt–Polymer-based FET biosensors exhibited high sensitivity and selectivity towards dopamine at unprecedentedly low concentrations (0.1 pM). Finally, a potentiometric FET sensor whose sensing element resides at the Au gate–aqueous solution interface by means of a self-assembled monolayer composed of cysteamine and 4-formylphenyl boronic acid was fabricated [151]. Even if this device did not contain any organic polymer but an organic self-assembled monolayer, it is considered in this section due to its very interesting sensing performances. Indeed, the covalent and selective adsorption of dopamine induces a surface dipole potential, which shifts the electrode work function and modulates the double-layer capacitance. As a result, this device is capable to detect dopamine up to pM concentration.

**Table 5:** A comparison of the performances of electrochemical dopamine biosensors based on conducting polymers.

Active Layer	Linear Range	Detection Limit	Reference
Polypyrrole	1–1000 µM	7 nM	[122]
Poly(pyrrole-3-carboxylic acid)	0.025–7.5 μM	2.5 nM	[123]
Poly(2-naphtol)	0.6–250 μM	95 nM	[130]
poly-4-amino-6-hydroxy–2-mercaptopyrimidine	2.5–25 μM	0.2 µM	[124]
Poly(eriochrome black T)	0.1–200 μM	20 nM	[127]
poly(safranine O)	0.3–10 µM	0.05 µM	[128]
poly(trypan blue)	1–40 µM	0.36 µM	[129]
poly(1,5-diaminonaphthalene)–SO_3_^-^	5–100 µM	0.1 µM	[131]
PEDOT–ferrocene	0.01–0.9 mM	1 µM	[133]
PANI–Au NPs	20–100 µM	16 µM	[135]
PEDOT–sodium dodecyl sulfate	0.5–140 µM	0.39 nM	[134]
PANI–Au NPs	10–1700 µM	5 µM	[136]
PEDOT–Graphene oxide	0.1–175 µM	39 nM	[137]
PANI–Graphene–aptamer	0.007–90 nM	1.98 pM	[138]
Polypyrrole–Graphene	0.8–10 µM	4 nM	[139]
PEDOT–CNT	0.1–20 µM	20 nM	[140]
Polycystine–CNT	10–200 µM	2.8 µM	[141]
Poly(anilineboronic acid) –CNT	1–10 nM	0.0.16 nM	[142]
PEDOT–nanoceria-MWCNT	0.1–400 µM	0.03 µM	[143]
PEDOT:PSS (FET)	50 nM–3 µM	5 nM	[146]
PEDOT:PSS (FET)	5–100 µM	6 µM	[147]
Carboxylated polypyrrole–CNT–aptamer (FET)	0.1 nM–10 µM	100 pM	[149]
3-carboxylate polypyrrole–Pt NPs	0.1 pM–1 nM	0.1 pM	[150]
cysteamine and 4-formylphenyl boronic acid (FET)	1 pM–1 mM	1 pM	[151]

#### 4.3.2. Molecularly Imprinted Polymers

Borrowing inspiration from the high affinity and specificity of biorecognition probes, such as antibodies and aptamers towards their target molecules, molecular imprinting technique has been developed to prepare a molecularly imprinted polymers (MIPs) with the purpose of acting as synthetic receptors for a targeted molecule [152]. Indeed, the MIP technique is a template-directed polymerization method in which a solution containing a template (target) molecule, a monomer, and a crosslinker, are dissolved in a suitable solvent, resulting in a crosslinked polymer. The polymerization step is followed by a template removal step, leaving permanent nanoscale cavities of the original template, which correspond to the shape, size, and orientation of target molecules. As a consequence, these polymers offer high selectivity and are very promising for electrochemical detection of proteins [153,154], antibiotics [155,156], hormones [157,158], or neurotransmitters such as dopamine, even though they can suffer from low sensitivity when they lack conductivity and electrocatalytic activity. That is why many of the MIPs used in biosensing are conductive or combined with conductive nanomaterials, such as gold or carbon nanomaterials.

Using this strategy, an electrochemical sensor was developed by the electropolymerization of conducting pyrrole onto a silica colloidal crystal template modified glassy carbon electrode surface in the presence of dopamine [159] (Table 6). Then, a 3D-ordered macroporous MIP electrochemical sensor was obtained by etching silica microspheres and extracting dopamine subsequently. Due to its high surface area, the prepared sensor provided much more efficient imprinted sites and exhibited fast-binding dynamics, good specific adsorption capacity, and high selective recognition to template molecule since peak current of dopamine varied linearly in the range of 2–0.23 mM with a detection limit of 0.9 µM. Similarly, another dopamine biosensor was prepared by deposition of a molecularly imprinted polymer film on the surface of electrodeposited hollow nickel nanospheres and o-phenylenediamine [160]. The use of 3D-nanospheres as a support material enlarged the sensing area and conductivity, while the MIP film warranted an improved selectivity. After optimization, this resulting biosensor showed high selectivity and a very low detection limit of 1.7 × 10^−14^ M.

Conducting polymer nanowires also offers great potential in the preparation of biosensors due to their large surface area and electrical conductivity. That is why 3D structures comprising polypyrrole nanowires and MIPs, obtained by electropolymerization of dopamine with o-phenylenediamine via cyclic voltammetry technique, were prepared by electropolymerization on the surfaces of a glassy carbon electrode [161]. The resulting biosensor possessed both large surface area and good electrocatalytic activity for oxidizing dopamine, and this leads to high sensitivity and a low limit of detection of 33 nM. Moreover, the electropolymerized MIP presents a large number of accessible surface imprints, increasing the biosensor selectivity. Similarly, a dopamine electrochemical biosensor based on MIP arrays modified over vertically aligned ZnO nanotubes was prepared [162]. First, ZnO nanorods were electrodeposited and transformed into ZnO nanotubes by chemical etching in KOH. Then, dopamine molecules were electropolymerized with pyrrole on both the inner and outer surfaces of the ZnO nanotubes. Due to the imprinted cavities that are always located at both the inner and the outer surface of ZnO, the ZnO nanotubes supported MIPs arrays display good accessibility towards template and can be used as biosensing materials for dopamine with high sensitivity, excellent selectivity, fast response, and large linear range (0.02–800 μM).

As previously presented, gold and carbon nanomaterials can also be combined with MIPs to increase the conductivity of these sensing materials. Thus, a dopamine-imprinted sensor in which gold nanoparticles were electrodeposited onto glassy carbon electrode, followed by self-assembly of the monomer p-thioaniline monolayer onto the electrode surface, through Au–S bonds formed between gold nanoparticles and thiol groups [163]. The amino groups of the polymer, necessary for the interaction with the dopamine through hydrogen interactions, were exposed by dipping in ethanol the sensor. This sensor demonstrated its limit of detection in the range of picomolar and showed good selectivity. Another biosensor was obtained by electropolymerization of p-aminobenzenethiol, followed by its doping with gold nanoparticles [164]. The resulting electrode was used for the amperometric detection of trace dopamine in human serums and exhibited high sensitivity and selectivity with a linear range going from 0.02 μM to 0.54 μM and a detection limit of 7.8 nM. Moreover, the imprinted polymer effectively avoided the interferences caused by ascorbic acid and uric acid, which coexist with dopamine in biological samples. In another work, a gold electrode was modified by biocompatible sulfonated graphene and by a dopamine-imprinted film polymerized in the presence of conducting o-phenylenediamine monomer [165]. This biosensor allowed the detection of dopamine in the range of 3–50 µM, and the low detection limit was 0.7 µM. The sensor also showed good selectivity towards dopamine due to the presence of electron-rich groups (sulfonate groups), which could bind effectively with cations present in dopamine and established excellent selectivity for dopamine against ascorbic acid. Another composite electrode was obtained by growing a nanometer thin film of ceramic-multiwalled carbon nanotubes, modifying it with benzyl N,N-diethyldithiocarbamate, followed by dopamine imprinting, under UV irradiation, in the presence of 4-nitrophenyl acrylate as functional monomer and ethylene glycol dimethyl acrylate as cross-linker, leading to imprinted cavities [166]. A wide linear concentration relationship, the excellent limit of detection (≈0.001 nM), and selectivity were obtained with this modified electrode using differential pulse anodic stripping voltammetric biosensing of dopamine in real biologic fluids. Another electrochemical biosensor using the molecularly imprinted oxygen-containing polypyrrole-decorated carbon nanotubes composite was developed for in vivo detection of dopamine [167]. This sensor exhibited a broad linear range of 5.0 × 10^−11^–5.0 × 10^−6^ M and the low limit of detection of 10 pM, which could be due to the high number of cavities for binding dopamine by the π–π stacking between the aromatic rings and by the hydrogen bonds between the amino groups of dopamine and the oxygen-containing groups of the polymer.

## 5. Conclusions and Perspectives

The detection of dopamine in real-life applications is a promising and exciting area of research, as indicated by hundreds of publications on this topic. Electrochemical biosensors are a promising technique for the detection of dopamine or other neurotransmitters. In this review, we summarized recent studies of nanomaterial-modified electrodes for the detection of dopamine. In recent years, most of the studies were devoted to the preparation of new sensing materials to improve biosensor performances. Thus, various nanomaterials (e.g., metals, metal oxides, carbon materials, polymers, and a combination of these materials) were reported to be excellent in improving both the sensitivity and the selectivity of modified electrodes. Specifically, in the case of electrochemical detection, the nanomaterials showed their ability to facilitate electron transfer reactions on the electrode, improving the detection of unique oxidation peaks or significant currents.

Now, modified electrodes have proved that they can be useful for dopamine detection in standard conditions and exhibited detection limits in the picomolar range, as well as selectivity towards dopamine even in the presence of interferents. It can be considered that they have shown a grade of maturity good enough to implement them in real analytical applications. Thus, the technology must now move from standard conditions to biomedical and clinical applications and be implanted in point-of-care devices. The future of dopamine biosensors probably consists of the development of miniaturized systems or platforms (consisting of arrays of nanoelectrode sensors or field-effect transistors) that must be portable, non-invasive, cheap, wearable, and capable of reducing the time and frequency of sampling. Thanks to the recent advances in miniaturized system technologies, it can be expected that future electrochemical biosensors will be capable of performing localized measurements of dopamine levels from living neurons that will facilitate the discovery of new types of drugs and techniques, which will ultimately contribute to the treatment of various dopamine-related diseases, including Parkinson’s disease, schizophrenia, Alzheimer’s disease, stress, and depression.

## Figures and Tables

**Table 2:** A comparison of the performances of electrochemical dopamine biosensors based on metal oxide nanomaterials.

Active Layer	Linear Range	Detection Limit	Reference
FePt–Fe_3_O_4_	0.1–90 µM	1 nM	[71]
NiO	0.5–5 μM	85 nM	[72]
MoS_2_	0.006–181 μM	2 nM	[73]
MoS_2_	10 pM–10 µM	2.3 pM	[74]
ZnO	0.1–800 µM	60 nM	[75]
Fe_3_O_4_	2–600 nM	0.8 nM	[76]
ZnFe_2_O_4_	2–600 nM	0.4 nM	[77]

**Table 3:** A comparison of the performances of electrochemical dopamine biosensors based on carbon nanotubes.

Active Layer	Linear Range	Detection Limit	Reference
SWCNT	1.2–900 µM	0.57 µM	[86]
MWCNT	1.2–800 µM	0.16 µM	[87]
Carbonyl–SWCNT	10–200 nM	15 nM	[88]
SDS–SWCNT	5–100 µM	20 nM	[89]
Nafion+poly(3-methylthiophene)-SWCNT	5–177 μM	2 µM	[90]
Ionic liquid–SWCNT	0.5–30 µM	0.16 µM	[91]
Polypyrrole–SWCNT	0.1–100 µM	136 pM	[92]
AgAu–MWCNT	3 nM–2.3 µM	0.23 nM	[93]
Graphene–MWCNT	5 nM–100 µM	0.87 nM	[94]
MoS_2_–MWCNT	0.03–1950 µM	13 nM	[95]

**Table 6:** A comparison of the performances of electrochemical dopamine biosensors based on molecularly imprinted polymers.

Active Layer	Linear Range	Detection Limit	Reference
Polypyrrole–SiO_2_	2 µM–0.23 mM	0.9 µM	[159]
Phenylenediamine–Ni	0.05–50 pM	0.017 pM	[160]
Polypyrrole/phenylenediamine	50 nM–100 µM	33 nM	[161]
Polypyrrole–ZnO	0.02–800 µM	1 nM	[162]
Poly(thioaniline)–Au NPs	1 nM–5 µM	33 pM	[163]
Poly(Aminobenzenethiol)–Au NPs	0.02–0.74 µM	7.8 nM	[164]
Phenylenediamine–Graphene–SO_3_^-^	3–50 µM	0.7 µM	[165]
Poly(nitrophenyl acrylate)–ceramic MWCNT	6.5–550 µM	1 mM	[166]
Polypyrrole–CNT	50 pM–5 µM	10 pM	[167]

## Data Availability

No new data were created or analyzed in this study. Data sharing is not applicable to this article.
